# Molecular Mechanisms of Obesity-Linked Cardiac Dysfunction: An Up-Date on Current Knowledge

**DOI:** 10.3390/cells10030629

**Published:** 2021-03-12

**Authors:** Jorge Gutiérrez-Cuevas, Ana Sandoval-Rodriguez, Alejandra Meza-Rios, Hugo Christian Monroy-Ramírez, Marina Galicia-Moreno, Jesús García-Bañuelos, Arturo Santos, Juan Armendariz-Borunda

**Affiliations:** 1 Department of Molecular Biology and Genomics, Institute for Molecular Biology in Medicine and Gene Therapy, University of Guadalajara, CUCS, Jalisco 44340, Mexico; gutierrezcj05@gmail.com (J.G.-C.); anasol44@hotmail.com (A.S.-R.); christian.monroy0981@gmail.com (H.C.M.-R.); marigamo_11@hotmail.com (M.G.-M.); bgarcia@cucs.udg.mx (J.G.-B.); 2 Tecnologico de Monterrey, Campus Guadalajara, Zapopan, School of Medicine and Health Sciences, Jalisco 45201, Mexico; alejandramezarios@yahoo.com.mx (A.M.-R.); arturo.santos@itesm.mx (A.S.)

**Keywords:** obesity, cardiovascular diseases, pathophysiology, PPARs, epigenetic modifications, gut microbiota dysbiosis, biomarkers, animal models, therapeutic treatments

## Abstract

Obesity is defined as excessive body fat accumulation, and worldwide obesity has nearly tripled since 1975. Excess of free fatty acids (FFAs) and triglycerides in obese individuals promote ectopic lipid accumulation in the liver, skeletal muscle tissue, and heart, among others, inducing insulin resistance, hypertension, metabolic syndrome, type 2 diabetes (T2D), atherosclerosis, and cardiovascular disease (CVD). These diseases are promoted by visceral white adipocyte tissue (WAT) dysfunction through an increase in pro-inflammatory adipokines, oxidative stress, activation of the renin-angiotensin-aldosterone system (RAAS), and adverse changes in the gut microbiome. In the heart, obesity and T2D induce changes in substrate utilization, tissue metabolism, oxidative stress, and inflammation, leading to myocardial fibrosis and ultimately cardiac dysfunction. Peroxisome proliferator-activated receptors (PPARs) are involved in the regulation of carbohydrate and lipid metabolism, also improve insulin sensitivity, triglyceride levels, inflammation, and oxidative stress. The purpose of this review is to provide an update on the molecular mechanisms involved in obesity-linked CVD pathophysiology, considering pro-inflammatory cytokines, adipokines, and hormones, as well as the role of oxidative stress, inflammation, and PPARs. In addition, cell lines and animal models, biomarkers, gut microbiota dysbiosis, epigenetic modifications, and current therapeutic treatments in CVD associated with obesity are outlined in this paper.

## 1. Introduction

The obesity epidemic has spread globally in the past four decades. Nowadays, more than a third of the world population is obese or overweight [1,2]. Obesity prevalence in children and young adolescents aged 5–19 years has increased from 4% in 1975 to 18% in 2016 [1]. In the United States, according to data reported in 2015 to 2016, the prevalence of obesity was 39.8% in adults, while in adolescents (12 to 19 years) was 20.6%, for children 6 to 11 years of age was 18.4%, and for children 2 to 5 years of age it was 13.9% [3]. In 2019, an estimated 38.2 million children under the age of 5 years had overweight or were obese [4]. Excess white adipocyte tissue (WAT), mainly visceral accumulation, is highly associated with dyslipidemia, systemic insulin resistance, hypertension, metabolic syndrome, obstructive sleep apnea, as well as type 2 diabetes (T2D), atherosclerosis, and cardiovascular disease (CVD) [2,5]. Globally, CVD is the main cause of death; an estimated 17.9 million people died in 2016, which represented 31% of all global deaths. Of these deaths, 85% were related to heart attack and stroke. One-third of these deaths occur prematurely in people under 70 years of age, mainly in low- and middle-income countries [6]. In addition, CVD-provoked death is two to three times more likely if patients have a body mass index (BMI) ≥ 35 kg/m^2^ [7], and a 30% increased risk of CVD associated mortality with each 5 kg/m^2^ increase in BMI [8].

Obesity is associated with a chronic inflammation that persists in the visceral adipose tissue (VAT) [9]. In this regard, adipose tissue inflammation and oxidative stress lead to reduced production of adiponectin as well as increased secretion of resistin, leptin, and pro-inflammatory adipokines and cytokines, thus contributing to cardiovascular stiffness, an impaired vascular relaxation, and finally to cardiac diastolic dysfunction [5]. Also, the renin-angiotensin-aldosterone system (RAAS) is activated as a result of obesity; this endocrine axis has considerable participation in the hemostasis of the cardiovascular system. Under pathophysiological conditions, RAAS stimulates inflammation and structural remodeling, thus inducing cardiac and vascular injury [5,10]. Different biochemical factors such as angiotensin II (Ang II), transforming growth factor beta (TGF-β), insulin-like growth factors (IGFs), and others modulate ECM production by cardiac fibroblasts. Fibroblasts in the heart perform the synthesis and deposition of ECM components, such as collagen I (Col I) and collagen III (Col III), which are the main elements of the connective tissue network [11,12,13]. Excessive collagen deposition, such as Col I, III, and IV, contribute to impaired left ventricular (LV) function in diabetic cardiomyopathy [14]. Several deleterious effects on the heart are induced by obesity and T2D, including changes in substrate utilization, tissue metabolism, oxidative stress, and inflammation; all of them are considered to promote left ventricular hypertrophy (LVH), fibrosis, arrhythmia, heart failure (HF)—particularly HF with preserved ejection fraction (HFpEF)—atrial fibrillation (AF), cardiac remodeling, and myocardial infarction, as well as diastolic dysfunction and LV systolic dysfunction [2,14,15].

Peroxisome proliferator-activated receptors (PPARs) play a critical physiological role in lipid regulation and carbohydrate metabolism, enhancing insulin sensitivity and restraining the inflammatory process and oxidative stress [16]. PPARα regulates several genes, particularly those related to lipids β-oxidation [17]. Meanwhile, PPARγ plays a critical regulatory role in glucose metabolism, adipocyte differentiation, and lipid storage through the regulation of genes with important participation in these metabolic processes [18]. Because of PPARs role in heart disease development, they are considered promising pharmacological targets for CVD treatment [19].

Many animal models and in vitro studies have been conducted to elucidate the mechanisms involved in cardiac injury and dysfunction during obesity. Mouse models are commonly used to study changes in cardiac tissue structure and function caused by high-fat diet (HFD)-induced obesity, yet, other animal models are used as well [20]. Biomarkers mainly denote biochemical changes at the tissue or organ level, and they are important to diagnosis or evaluation of diseases in the development of treatments and are considered surrogate endpoints for clinical trials [21].

In recent years, the gut microbiota has demonstrated its essential role for the human host, modulating glucose and lipid homeostasis, regulating satiety, vitamin production, and metabolites with important roles in physiological functions [22,23]. Also, high blood pressure is associated with dysbiosis in the gut microbiota in both rat models and human hypertension [24]. Additionally, heart insults induced by obesity may produce DNA methylation changes, and several studies have suggested that histone methylations are implicated in cardiac hypertrophy and failure [25,26]. Histone deacetylases (HDACs) have been implicated in the pathogenesis of fibrosis (in models of both hypertrophic and ischemic heart disease) and in cardiac remodeling in the settings of pressure overload and ischemia/reperfusion [27].

This review provides an update on the pathophysiology of obesity-associated CVD, including the role of PPARs, inflammatory and oxidative processes, gut microbiota dysbiosis, and offers concise information about epigenetic modifications in CVD. Likewise, this manuscript provides information on some experimental models used to study CVD and useful biomarkers for the diagnosis and prognosis of this disease. Finally, it does address several therapeutic approaches for the treatment of CVD induced by obesity.

## 2. Pathophysiology of Obesity-Induced Cardiovascular Disease

### 2.1. Cardiac Adipose Tissue Associated with CVD

The heart can allocate fat in three compartments: epicardial adipose tissue (EAT), paracardial adipose tissue (PAT), and pericardial fat [5]. In CVD development, perivascular adipose tissue is important; it includes EAT adjoining coronary arteries and fatty deposits around the aorta and the medium and small arteries. Moreover, the expression of various vasoconstrictors such as resistin, Ang II, and chemerin is increased in perivascular adipocytes [9]. The amount of EAT is strongly correlated with visceral obesity [9]. EAT local negative effects in the myocardium include natural compression, local delivery of free fatty acids (FFAs) and cardioactive hormones, and the release of pro-inflammatory adipokines, resulting in cardiac maladaptive morphologic and dysfunction changes. Furthermore, an increase in EAT also causes coronary calcification, atheromatous plaque formation, and coronary artery disease (CAD) [5,28]. Ng et al. reported that increased EAT volume index and insulin resistance were independently associated with increased myocardial fat content and interstitial myocardial fibrosis. Augmented EAT volume also was associated with a diminution in LV global longitudinal strain [29]. EAT thickness, or PAT volume, was associated with low high-density lipoprotein (HDL) levels, an increase in fasting glucose, higher C-reactive protein (CRP), and other cardiovascular risk characteristics. It is important to note that most of these correlations were removed after adjusting for VAT mass [28]. PAT mass significantly correlates with hypertension, elevated triglycerides, and decreased HDL, but after VAT adjustment, this correlation disappeared. However, epidemiological studies showed an association between PAT volume and calcium deposition in coronary arteries, even after adjustment for VAT [28]. Therefore, excess body fat content is involved in the pathogenesis of CAD and CVD.

### 2.2. Effects of Adipokines in CVD

Adiponectin, an adipokine mainly secreted from WAT, is diminished in obesity due to both decreased adiponectin secretion and reduced receptor expression [30]. Furthermore, in obese patients affected with CAD, production of adiponectin by EAT is decreased [9]. Adiponectin is inversely correlated with cardiovascular risk factors such as hypertension, atherosclerosis, dyslipidemia, and hyperglycemia and is a potential therapeutic target for diastolic dysfunction. In the heart, adiponectin protects and prevents myocardial hypertrophy, cardiac fibrosis, atherosclerosis, inflammation, nitrative and oxidative stress, and angiogenesis [14]. Additionally, adiponectin antagonizes the actions of endogenous vasoconstrictors, including the activity of renal sympathetic nerves, and induces natriuresis by inhibiting the secretion of aldosterone. Adiponectin ameliorates the capacity of the heart to sustain pressure or volume overload and protects the heart against ischemic injury [31].

On the other hand, leptin is an adipokine that regulates appetite and body fat mass mostly through the central nervous system. Adipocyte-release of leptin is directly correlated with fat deposition. The heart expresses high amounts of leptin receptors, and cardiomyocytes can release leptin. In murine models, mutations in leptin or its receptor conduce to altered metabolism in cardiomyocytes, cardiac steatosis, and cardiac dysfunction [31]. Obese patients often have elevated levels of leptin and are resistant to its actions on the central nervous system to inhibit food intake. In obesity, leptin-mediated increases in aldosterone promote sodium retention, increase cardiac filling pressures, exacerbate remodeling, and accelerate the progression of HF. The interaction of leptin with Ang II and mineralocorticoid receptors facilitate the inflammatory process and can cause cardiac hypertrophy and fibrosis. Furthermore, both increases in leptin and a diminution of adiponectin signaling likely contribute to obesity-related HFpEF [31].

In mice fed HFD, autotaxin accumulation was associated with cardiac dysfunction in obese mice. On the other hand, autotaxin blockade protected obese mice against structural cardiac disorders, hypertrophy, and LV dysfunction [32].

Neprilysin is not considered an adipokine; however, it is expressed on the surface of mature adipocytes. People with obesity have elevated levels of neprilysin. This molecule degrades endogenous natriuretic peptides, inhibiting renal sodium reabsorption, suppressing aldosterone secretion from the adrenal gland, in addition to inhibiting inflammation and fibrosis [31]. In obese subjects with HFpEF, soluble neprilysin levels are high; its inhibition, on the contrary, reduces ventricular overload and improves LA overfilling in these patients [31]. Therefore, adipokines play an important role in the protection of cardiac dysfunction.

### 2.3. The Role of Oxidative Stress in CVD Pathogenesis

There is data about the connection between obesity, oxidative stress, and an increase in HF. Hyperglycemia and insulin resistance increase reactive oxygen species (ROS) production and promote inefficiency in the antioxidant systems in obese rats [33]. In young patients with obesity, this condition promotes disturbed mitochondrial function, ROS release, and cell death [34]. Moreover, cardiac steatosis generates several lipotoxic intermediates such as acylcarnitine, diacylglycerol (DAG), and ceramides. It was reported that DAG and ceramides generate ROS, and ceramides increase ROS production through disruption of mitochondrial electron transport chain, thereby inducing apoptosis and insulin resistance. DAG induces insulin resistance via protein kinase C (PKC) signaling, which inhibits insulin receptor substrate 1 (IRS-1) phosphorylation. Furthermore, ROS increases some oxidative stress markers such as 8-hydroxy-2-deoxyguanosine (8-OHdG) and protein carbonyl, and possibly reduced cell viability [35,36]. In the heart, cardiac myocytes, endothelial cells, and neutrophils are sources of ROS through NADPH oxidase overactivity, allowing myocardial remodeling, including contractile dysfunction and structural alterations [37,38]. ROS cause these cardiac alterations by the following mechanisms: (1) ROS activate a broad variety of hypertrophy signaling kinases and transcription factors, such as tyrosine kinase Src, regulating smooth muscle function through of the control of actin-cytoskeleton dynamics [39]; GTP-binding protein Rac, associated with hypertrophy and smooth muscle cell proliferation, endothelial dysfunction, as well as hypertension, and atherosclerosis [40]; mitogen activated protein kinase (MAPK), and c-Jun N-terminal kinase (JNK), related to cell growth, differentiation, development, the cell cycle, survival and cell death; and the nuclear factor-kappa B (NF-κB) pathway, related to pro-inflammatory gene transcription [41,42]; (2) ROS promote apoptosis through to apoptosis signal regulating kinase 1 (ASK1) modulation; (3) ROS cause DNA strand breaks via activating of nuclear enzyme poly(ADP-ribose) polymerase-1 (PARP-1), allowing survival and cell death regulation; (4) ROS can activate matrix metalloproteinase (MMPs), which are increased in the failing heart; and (5) ROS directly affect contractile function by modifying proteins implicated in excitation-contraction coupling such as sarco/endoplasmic reticulum Ca^2+^ ATPase (SERCA) [37,41], thus leading to cardiac dysfunction.

Leptin is a mediator of cardiac alteration in obesity, specifically can exert pro-fibrotic and pro-oxidant effects through the activation of PI3k-Akt signaling pathway, and subsequently the activation of TGF-β and connective tissue growth factor (CTGF) [43,44]. Studies carried out in rats fed HFDs have shown that leptin locally leads to heart alterations associated with obesity through induction of collagen production, which is mediated by oxidative stress and by the mTOR signaling pathway [44]. Other evidence of the relationship between oxidative damage and CVD development is the nuclear factor erythroid-2 related factor (Nrf2), an important regulator of redox signaling that acts as a transcriptional activator of antioxidant response elements (ARE)-responsive genes such as hemeoxygenase-1 (HO-1), glutathione-S-transferase (GST), glutathione peroxidase (GPx), NAD(P)H quinone oxidoreductase 1 (NQO1), superoxide dismutase (SOD), catalase (CAT), and glutathione reductase (GR), just to name a few [45]. Some studies have demonstrated that Nrf2-knockout mice develop cardiac hypertrophy, while activation of this nuclear factor by specific pharmacological activators such as epigallocatechin 3-gallate is effective to induce the expression and activation of Nrf2 in adipose tissue of obese mice, improves lipidemic control, and decreases the oxidative process, which could improve cardiovascular function [45,46]. Additionally, oxidative stress causes cardiac hypertrophy via oxidation of cysteines in class II histone deacetylases, which are master negative regulators of hypertrophy [47]. This evidence suggests that the oxidative process plays an important role in the pathogenesis of CVD, and antioxidant therapy may be a suitable option for the treatment of this disease.

### 2.4. Inflammation and Fibrosis in the Development of CVD

In obesity-generated insulin resistance, WAT expresses increased levels of pro-inflammatory and proliferative adipokines, including leptin, dipeptidyl peptidase 4 (DPP-4), visfatin, toll-like receptor-4, resistin, MCP-1, TNF-α, IL-6, and IL-8, causing deterioration of the metabolic signaling of insulin and cardiovascular dysfunction [5]. In addition, some studies have demonstrated that the FFAs, particularly palmitic acid, can activate the signaling pathway of NF-κB, increasing the expression of pro-inflammatory cytokines such as TNF-α, IL-6, and IL-1β through the TLR4/MyD88 signaling pathway [48,49]. The TLR4/MyD88 signaling pathway has an important role in the development of metabolic disorders, including CVDs [48,50]. Moreover, DAG also induces inflammation by activating NF-κB and altering Ca^2+^ handling [41].

When cardiac damage occurs, as seen in obesity-related stretching or diabetes-related glycosylation, fibroblasts differentiate into myofibroblasts and acquire pro-fibrotic and pro-inflammatory properties [2,51]. Myofibroblasts can be activated by advanced glycation end products (AGEs) with or without TGF-β, and this response involves the AGE receptor (RAGE) and extracellular-signal-regulated kinases (ERK)1/2 activation [52]. Activated cardiac myofibroblasts produce pro-inflammatory cytokines (interleukin-1 (IL-1), IL-6 and TGF-β), vasoactive proteins (Ang II, endothelin-1 (ET-1), atrial natriuretic peptide (ANP) and B-type natriuretic peptide (BNP)), noradrenaline, ischemia, reperfusion, and mechanical stimuli [52]. With respect to IL-6, it has fibrogenic actions, and its pro-fibrogenic response is related to STAT3-stimulation, which leads to collagen production by cardiac fibroblasts or by TGF-β stimulation [53]. In vitro studies show that IL-11, another member of the cytokines IL-6 family, plays a critical role in the pathogenesis of fibrosis, and its inhibition alters the activation of fibroblasts induced by TGF-β [54]. With respect to TNF-α, it can induce fibrosis acting on cardiac fibroblasts [55,56]. Additionally, monocytes and macrophages also have an important role in pro-inflammatory mediators production, such as cytokines and pro-fibrogenic growth factors [57]. In an early inflammation stage, monocytes with pro-inflammatory, phagocytic, and proteolytic properties are recruited, and there is an expression of CCR2 chemokine receptors [58]. On the other hand, TGF-β stimulates the activation of myofibroblasts by up-regulating α-SMA expression [2]. Our group demonstrated that in the ventricular tissue of mice subjected to a model of nonalcoholic steatohepatitis (NASH) induced by high fat and sugar diet, an up-regulation in α-SMA and Col I and III, among other mRNAs, takes place. In addition, cardiac hypertrophy and fibrosis were found [59]. Activation of the RAAS in WAT leads to insulin resistance through activation of the mTOR/S6K1 signaling pathway, in addition to increasing oxidative stress [5]. mTOR induces the activation of glucocorticoid-regulated kinase 1 (SGK1) and epithelial sodium channel, inducing fibrosis in adipose, cardiovascular, and renal tissue. These pathophysiological processes decreased endothelial nitric oxide synthase (eNOS) activation and nitric oxide (NO) bioavailability in association with increased cardiovascular stiffness and impaired relaxation [5]. Excess aldosterone is closely associated with systemic inflammation, endothelial dysfunction, arterial stiffness, hypertension, and cardiac hypertrophy [60]. In summary, pro-inflammatory adipokines secreted by EAT and WAT modify the normal electromechanical changes in atrial tissues, left atrial (LA) enlargement, and cardiac remodeling (characterized by fat accumulation, fibrotic infiltration, and hypertrophy), increasing the risk of AF, which is the most common form of arrhythmia [2,5,28]. Meanwhile, TGF-β, leptin, and Ang II are potent stimulators of collagen synthesis, thus causing cardiac, pericardial, and vascular fibrosis. Together, these alterations in the ECM cause abnormalities in cardiac contraction, relaxation, and conduction, leading to HF [2,31]. The use of pharmacological molecules with the ability to modulate the signaling pathways discussed may be an important strategy for the treatment of CVD.

### 2.5. Left Ventricular Hypertrophy

In obesity-generated insulin resistance, left ventricular hypertrophy (LVH) is defined as an increase in LV mass [30]. LVH occurs in two ways, especially in severe obesity; (1) concentric hypertrophy, which is caused by chronic pressure overload and leads to decreased LV volume and augmented wall thickness, and (2) eccentric hypertrophy that originates from volume overload and generates dilation and thinning of the heart wall [28,61]. Cardiomyocyte hypertrophy is a consequence of FFA accumulation and lipotoxicity. Furthermore, fat invasion impairs cardiac contractility and restricts the dilating capacity of the left ventricle [30]. Our group reported cardiomyocyte hypertrophy in mice with NASH induced by a high-fat/high-carbohydrate diet [59]. Glucotoxicity affects cardiomyocytes, and impaired insulin regulation raises Ang II, leading to myocardial hypertrophy, fibrosis, and apoptosis [30]. Hyperinsulinemia also activates the sympathetic nervous system, which promotes myocardial dysfunction [30].

Decreased adiponectin activity contributes to LVH through insulin resistance but also lost its effects in inflammation reduction, endothelial cell adhesion prevention, and decreasing foam cell accumulation in the heart. Meanwhile, hyperleptinemia promotes unfavorable cardiac sequelae, including elevated ROS in the heart, cardiomyocyte apoptosis, and direct induction of cardiac hypertrophy [30]. All members of natriuretic peptides family ANP—also known as atrial natriuretic factor (ANF)—BNP and C-type (CNP) have the ability to affect cardiovascular and endocrine systems through their actions over diuresis, natriuresis, vasorelaxation, as well as aldosterone and renin inhibition. Under hypertrophic conditions, ANP and BNP inhibit myocardial hypertrophy [61].

Data from a meta-analysis of 22 echocardiographic studies showed a relationship between obesity and LVH. In addition, a cardiac magnetic resonance study suggested a predominant concentric hypertrophic pattern in obese men and both concentric and eccentric hypertrophic in obese women [15]. Additionally, LVH and diastolic dysfunction are present in obese normotensive children. In adults, LVH is linked with ventricular arrhythmias and HF, conferring a four-fold risk of CVD morbidity and mortality [30].

### 2.6. Hemodynamic Alterations

Obesity, particularly visceral adiposity, can be linked with three different phenotypes of HF, (1) HF with a reduced ejection fraction, (2) HF with HFpEF, and (3) high-output HF. All these phenotypes are characterized by a high secretion of aldosterone and sodium retention [31]. In the first type of HF, obesity is commonly associated with a mild decrease in systolic function. In HF with HFpEF, if systolic function is preserved, but the distensibility of the heart is impaired due to inflammation or fibrosis, then sodium preservation and plasma volume expansion induce cardiac overfilling rather than cardiac dilatation. Myocardial, pericardial, and vascular fibrosis increased ventricular and aortic stiffness, explaining why cardiac chambers are only modestly enlarged in elderly people with obesity and HF. High-output HF occurs when the heart is able to undergo significant ventricular enlargement. Sodium retention associated with obesity and plasma expansion can lead to marked cardiac dilation with a normal systolic function. The heart can accommodate and expel the large volume of blood it receives; this results in a high output state with higher cardiac filling pressures [31].

Additionally, alterations of right heart hemodynamics, particularly the elevation of the pulmonary artery and right atrial pressures, can be found in extremely obese subjects. However, these findings are not typical in the asymptomatic stage [28].

### 2.7. Diastolic Dysfunction

Diastolic dysfunction occurs by alteration in ventricular relaxation, distensibility, or filling [14]. Abnormalities of LV diastolic performance related to metabolic diseases are characterized by delayed relaxation, with elevated LV filling pressure being less common. Nevertheless, the utilization of conventional Doppler for LV filling evaluation and LA enlargement may be somewhat problematic in overweight subjects, in whom the effects of increased loading can be an impediment to appropriate interpretation of results [28].

Several studies have reported mild diastolic dysfunction in obese subjects. This involved different echocardiographic measures such as prolonged LV relaxation time, augmented E/e ratio, and lower E/A ratio, suggestive of diastolic filling alterations and increased filling pressures. In addition, the prevalence of diastolic dysfunction augments with the gravity of obesity [15].

### 2.8. Systolic Dysfunction 

**Figure 1:**
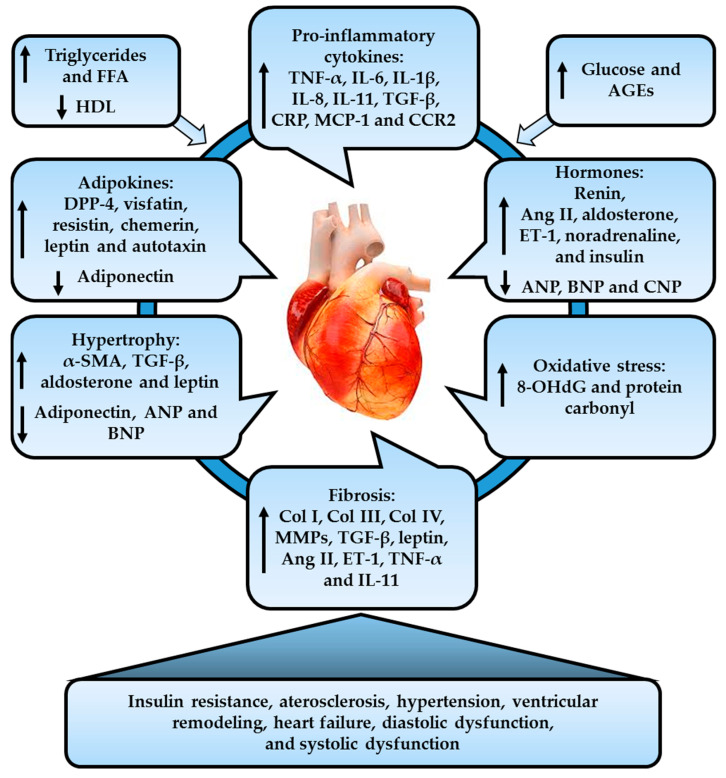
Pathophysiology involved in cardiovascular disease (CVD). Obesity-related cardiac tissue injury induces dysregulation in the expression of pro-inflammatory cytokines, adipokines, and hormones, leading to oxidative stress, inflammation, systemic insulin resistance, atherosclerosis, as well as cardiac fibrosis and hypertrophy, and ultimately cardiac dysfunction. FFA: free fatty acids; HDL: high-density lipoprotein; CRP: higher C-reactive protein; MCP-1: monocyte chemotactic protein 1; CCR2: chemokine re-ceptor 2; AGEs: advanced glycation end products; DPP-4: dipeptidyl peptidase 4; ET-1: endo-thelin-1; OHdG: 8-hydroxy-2-deoxyguanosine; MMPs: matrix metalloproteinase; ANP: atrial natriuretic peptide; BNP: B-type natriuretic peptide; CNP: C-type natriuretic peptide.

LV systolic function, assessed with LV ejection fraction by standard echocardiography, is normal or supranormal in obese individuals. Other studies using novel echocardiographic techniques showed subclinical systolic contractile abnormalities on tissue velocity and deformation in obese individuals without coronary or structural heart disease. Furthermore, these obese subjects showed a reduced spectral pulsed-wave systolic velocity, as well as a decreased regional and global tension, but the LV ejection fraction remained in a normal range [15]. Several studies have shown slightly decreased LV systolic function in obese and diabetic rats, transgenic and obese mice with HFD-induced insulin resistance, and transgenic mice with cardiac steatosis. However, in sheep with obesity induced by a high-calorie diet, LV systolic function verified by LV ejection fraction was not altered [15]. Dyslipidemia and hyperglycemia effects on the cardiac tissue in obese individuals are illustrated in Figure 1.

## 3. Peroxisome Proliferator-Activated Receptors, Keys Modulator in the Cardiac Fibrosis Process

Peroxisome proliferators are molecules with pleiotropic functions such as an increase in the number of peroxisomes, β-oxidation, and hypolipidemia; all these effects are regulated by the PPARs, and in the cardiac tissue are expressed in endothelial cells, vascular smooth muscle cells, and macrophages [62]. In the heart, the main role of PPARs is specifically β-oxidation and mitochondrial bioenergetics, which makes them a promising therapeutic target for cardiac disease treatment. Several studies have reported the biological roles of PPARs in CVDs, including cardiac hypertrophy and HF [62,63].

The family of PPARs is mainly composed of three isoforms: PPARα, PPARβ/δ, and PPARγ, each one with a specific tissue distribution pattern. PPARα is expressed in tissues with a high oxidative capacity and energy consumption, such as the heart and liver. PPARγ is expressed in adipose tissue or in some conditions of liver damage; finally, PPARβ/δ is more ubiquitous, expressed in the heart and skeletal muscle, and intestine [64,65].

PPARs can be activated by many endogenous ligands, such as long-chain fatty acids and eicosanoids, binding with different affinity to these receptors [66]. Moreover, many synthetic ligands have been designed for the different isoforms of PPARs to postulate them as therapeutic targets in the treatment of various chronic degenerative diseases [67]. Gene transcription regulated by PPARs is carried out when agonists are coupled to the ligand-binding domain of each PPAR, inducing heterodimerization with other members of the nuclear receptor superfamily, such as the retinoic X receptor, which binds to a sequence of repetitions known as the PPAR response element (PPRE) [68].

PPARα regulates a significant number of genes, mainly those related to the metabolism of fatty acids, particularly in the β-oxidation pathway such as carnitine palmitoyltransferase I, acyl-CoA oxidase, thiolase, sterol 12-hydroxylase (CYP8B1), fatty acid transport protein (FATP), fatty acid translocase (FAT/CD36), lipoprotein lipase, lipoprotein lipase (LPL) and apolipoprotein A-1 and A-II [17]. On the other hand, PPARγ plays an essential regulatory role in glucose metabolism, adipocyte differentiation, and lipid storage by controlling the transcription of several genes involved in these metabolic processes. Some key target genes of PPARγ include the fat-specific adipocyte protein 2 (aP2;-FABP), LPL, FAT/CD36, FA transport, FA-binding protein, acyl-CoA synthase, glucokinase, glucose transporter type 4 (GLUT4), phosphoenol pyruvate carboxykinase, uncoupling proteins (UCP) 1, 2, and 3, and the liver X receptor (LXR) [18].

**Figure 2:**
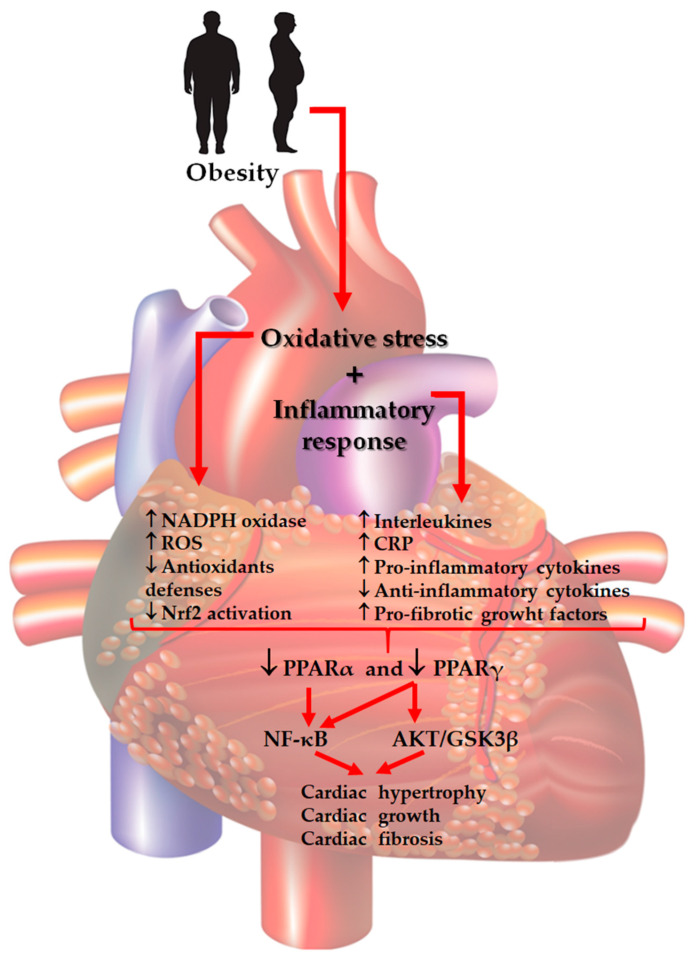
Main responses induced by oxidative damage and the inflammatory process, and involving peroxisome proliferator-activated receptors, PPARα and PPARγ, during the obesity-related cardiac fibrosis process. CRP: higher C-reactive protein; AKT: protein kinase B; GSK3β: glycogen synthase kinase 3 beta; NADPH: nicotinamide adenine dinucleotide phosphate reduced; ROS: reactive oxygen species.

In cardiac tissue, PPARs have several functions beyond their characteristic roles, these functions including extracellular matrix remodeling, oxidative stress, and inflammation. Regarding this, there exists strong evidence that PPARα activation is necessary to prevent cellular oxidative damage; therefore, a chronic inactivation of the PPARα signaling pathway may upset the balance between oxidant products and antioxidant defenses, allowing cardiac damage [19]. In *Pparγ* knockout mice, it was demonstrated that this nuclear factor plays an important role in cardiomyocytes and has the ability to prevent myocardial ischemia-reperfusion damage by modulating NF-κB function and subsequently inflammation response [69]. The role of PPARα in the myocardium has been elucidated in *Pparα*-/- knockout mice, demonstrating a reduced cardiac function [70]; this response is associated with structural defects in mitochondria, and consequently, an increase in oxidative damage [71]. The PPARα agonists were developed to treat dyslipidemia, for example, fibric acid derivatives and fibrates, which retards the development of atherosclerosis in ApoE-/- and LDLR-/- mice [72]. The drug fenofibrate exerts some PPARα-dependent and independent actions in microvascular endothelial cells, reducing ET-1 expression [73]. Furthermore, there is evidence that fenofibrate is more effective in patients with high triglyceride levels and low HDL-cholesterol. However, the mechanisms have not been entirely elucidated. Other drugs, such as gemfibrozil, reduced cardiovascular events, including coronary heart disease, myocardial infarction, and stroke in T2D patients, in a clinical study [72]. In addition, PPARα agonists such as clofibrate, and bezafibrate, as well as synthetic ligands of PPARγ, which include the thiazolidinedione drug class (rosiglitazone and pioglitazone), have been shown effective options for CVD treatment associated with metabolic diseases. However, they have several side effects that limit the safe use of these drugs [72]. It is important to mention that new PPARs agonists are currently being developed, which have few side effects, and could be an alternative treatment option for CVD. Recently, we demonstrated that prolonged-release pirfenidone (PR-PFD) is an agonist for PPARα [74], and PR-PFD reduces cardiac fibrosis in a mouse NASH model [59]. Therefore, the mechanisms of action of PPARs are versatile with a therapeutic potential to treat CVD and other metabolic diseases. In summary, the activation of PPARα prevents oxidative damage, while the activation of PPARγ modulates the inflammatory response by NF-κB. The challenge will be to design therapeutic strategies based on activation of PPARs, but with minimal side effects. Figure 2 illustrates the ways by which oxidative stress, inflammation, and the role of PPARs participate in obesity-related CVD development.

## 4. Epigenetics of Obesity-Linked Cardiac Dysfunction

**Figure 3:**
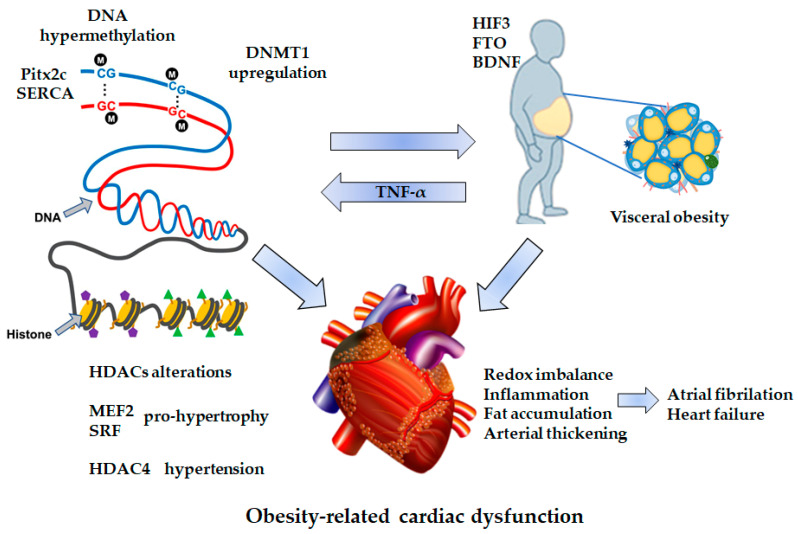
DNA and histone modifications in obesity-linked cardiac dysfunction. Visceral obesity causes systemic chronic inflammation and deregulation in hypoxia inducible factor 3 (HIF3), fat mass and obesity-associated protein (FTO), and brain derived neurotrophic factor (BDNF) gene methylation, as well as serum TNF-α increase. These events up-regulate DNMT1 and, in consequence, lead to DNA hypermethylation. Specifically, increased methylation in Pitx2c and sarco/endoplasmic reticulum Ca^2+^ ATPase (SERCA) promoters provoke a decrease in expression, contributing to heart failure pathophysiology. HDAC4 is a master negative regulator of cardiac hypertrophy, where it is found oxidized, triggering de-repression of pro-hypertrophy genes like myocyte enhancement factor 2 (MEF2) and serum response factor (SRF). These molecular mechanisms lead to atrial fibrillation and heart failure. HDACs: histone deacetylases; HDAC4: histone deacetylase 4; DNMT1: DNA methyltransferase 1; Pitx2c: paired-like homeodomain transcription factor 2, isoform c.

Multiple epigenetic processes, including DNA methylation, histone modification, and the expression of non-coding RNA molecules, affect gene expression, influencing the health and adaptability of the organism. Epigenetic changes are heritable and can be maternally and paternally transmitted to the offspring [75]. Hence, an unhealthy lifestyle influences not only our epigenome but those of our descendants. However, environmental exposure and lifestyle can also define epigenetic patterns throughout life. Epigenetic modifications are reversible, different among cell types, and can potentially lead to disease susceptibility by producing long-term changes in gene transcription [76]. Epigenetic modifications are potent modulators of gene transcription in the vasculature and might significantly contribute to the development of obesity-induced endothelial dysfunction, altering transcriptional networks implicated in redox homeostasis, mitochondrial function, vascular inflammation, and perivascular fat homeostasis [77]. Obesity-related vascular dysfunction is characterized by increased collagen deposition within the vascular wall, inflammatory infiltrate, perivascular fat accumulation, and progressive arterial thickening; ultimately, an augmented arterial stiffness in large vessels and a reduced lumen ratio in vessels. Obesity causes functional, morphological, and metabolic cardiac abnormalities, leading to HF or AF [20,70]. Epigenetic mechanisms implicated in the development of metabolic cardiomyopathy are described in Figure 3.

### 4.1. DNA Methylation Changes in Obesity-Related CVD

DNA methylation is the covalent attachment of a methyl group to the C5 position of cytosine, usually in CpG rich regions. The methyl groups may physically block transcription factors binding to DNA, or they can act as a binding site for transcriptional repressors, such as histone deacetylases. DNA methyltransferase 1 (DNMT1) is responsible for the recognition of the hemimethylated dsDNA following mitosis and for the methylation of the daughter strand. DNMT3a and DNMT3b are responsible for *de novo* methylation during embryogenesis, establishing new methylation patterns specific to each cell type [71]. The ten-eleven translocation (Tet) enzymes can remove the methyl group in DNA [78].

Perturbation of the redox-sensitive hypoxia inducible transcription factor (HIF) signaling has been found to be pivotal in the regulation of weight gain. Hypoxia inducible factor 3 subunit alpha (HIF3A) showed pronounced methylation in adipose tissue and blood cells associated with BMI [79]. Also, in the umbilical cords of infants, the first intron of the *HIF3* gene showed higher methylation in three CpGs that were associated with adiposity and greater infant weight [80]. Fat mass and obesity-associated protein (FTO) is an alpha-ketoglutarate dependent dioxygenase that acts as a regulator of fat mass, adipogenesis, and energy homeostasis. Mansego et al. showed that methylation levels of *FTO* and brain derived neurotrophic factor (*BDNF*) were associated with body weight gain and body weight [81]. On the other hand, Kao et al. showed that TNF-α, a pro-inflammatory cytokine, was involved in heart diseases and obesity, directly enhancing cardiac methylation through the up-regulation of DNMT1 [82]. Moreover, Ang II may increase the expression of DNA methyltransferase in arteries, and the inhibition of DNA methyltransferase could be important to avoid vascular wall thickness [83]. Correspondingly, Ang II-treated cardiomyocytes cell line exhibited reduced expression of Pitx2c protein associated with a 20% enhanced expression of DNMT1 protein. Pitx2c controls the growth of pacemaker cells in the left atrium and regulates cardiac sodium flow, conduction velocity, and resting membrane potential in cardiomyocytes. HF increases cardiac DNA methylation in the *Pitx2c* promoter downregulating Pitx2c by 50% [84]. Another mechanism involved is related to calcium regulation. SERCA dysfunction causes HF and increases the occurrence of cardiac arrhythmia. SERCA plays a critical role in calcium re-uptake after calcium-induced calcium release. *SERCA2a* promoter is enriched with CpG islands. In TNF-α-treated cardiomyocytes, methylation of the *ATP2A2* gene promoter is increased by three times with decreased expression of the protein, impairing calcium regulation [82]. Hypermethylation contributes to the pathophysiology of HF. Activation of RAS enhanced oxidative stress, and increased circulatory TNF-α in HF can predispose to DNA hypermethylation. Both animal and human studies have found a higher occurrence of DNA methylation in HF cardiomyocytes compared to normal hearts [85,86]. Movassagh et al. identified three angiogenesis-related genes (*AMOTL2*, *ARHGAP24*, and *CD31*) that were differentially methylated in human HF [87].

### 4.2. Role of Histone Modifications in Obesity-Linked Cardiomyopathy 

Histone modifications control the accessibility of nucleosomes for transcription and influence the binding capacity of other proteins to histones through changes in local hydrophobicity. Histones are subject to methylation, acetylation, phosphorylation, ADP-ribosylation, ubiquitination, and SUMOylation, among other modifications. Acetylation neutralizes the positive charge of the lysine in histones, weakening the bound with negatively charged DNA and releasing chromatin for gene transcription. Histone acetylates (HATs) catalyze histone acetylation, a reaction reversed by histone deacetylases (HDACs). Instead, histone methylation plays a role in gene expression that can induce opposite transcriptional patterns depending on which lysine residue is being methylated. For example, methylation of lysine 4 on histone 3 (H3K4) is an established marker for gene transcription, while methylation of H3K9 is known to be a repressive marker [88].

The mechanisms whereby HDACs modulate cardiac function are complex; some HDACs display antihypertrophic properties, whereas others exhibit pro-hypertrophic effects. For example, loss of function of HDAC5 or HDAC9 has been associated with binding and silencing myocyte enhancer factor 2C (MEF2C), leading to a higher susceptibility to cardiac hypertrophy and cardiac failure [89]. In contrast, HDAC4 (a master negative regulator of cardiac hypertrophy) was found to repress the activities of MEF2 and serum response factor (SRF) under physiological conditions, but in cardiac hypertrophy became oxidized, causing it to shuttle out of the nucleus and allow de-repression of pro-hypertrophy genes [47]. In experimental studies, HDAC inhibitors benefit the cardiac suppression of oxidative stress and inflammation, inhibiting MAP-kinase signaling and enhancing the clearance of protein aggregate and autophagic flux [90]. Insights into the mechanism include that HDACs are part of a chromatin repressor complex, which inhibits transcription of myosin heavy-chain-associated RNA transcripts (Mhrt); a long noncoding RNA, which protects the heart against pathological hypertrophy [91]. Additionally, modulation of inflammation by HDACs has important implications for cardiac diseases. In an experimental study, drug-induced HDAC inhibition attenuates cardiac hypertrophy and fibrosis and improved cardiac function [92]. Besides, experimental deletion of HDAC9 resulted in an atheroprotective effect due to increased accumulation of total acetylated H3 and H3K9 at the promoters of ATP-binding cassette transporter (ABCA1), ATP Binding Cassette Subfamily G Member 1 (ABCG1), and PPARγ in macrophages [93]. In a model of spontaneously hypertensive rats, HDAC4 demonstrated pro-inflammatory effects that mediate the further development of hypertension [94]. In summary, global methylation occurs at certain specific gene regions or specific histone positions and affects the transcription and expression of critical regulatory genes, which play key roles in arterial endothelial cell dysfunction, redox imbalance, cardiac fat accumulation, and inflammation in obesity-linked cardiomyopathy.

## 5. Gut Microbiota of Obesity Associated with CVD

The term microbiota refers to the assemblage of microorganisms living in a specific environment. The human microbiota is composed of bacteria, viruses, fungi, and other single-cell organisms colonizing different anatomic areas [95]. Around 80% of the human microbiota in healthy adults is represented by Firmicutes and Bacteroidetes, including Actinobacteria, Proteobacteria, Verrucomicrobia, and Fusobacteria, among other phyla [96]. Scientific evidence supports that changes in microbiota can promote diseases, including metabolic diseases like obesity, lipid disorders, T2D, and CVD. Mechanisms include insulin resistance, inflammation, vascular and metabolic impairment [23,96]. Increased IBM is associated with a higher risk for coronary diseases, suggesting a link between the gut and the heart [97]. 

**Figure 4:**
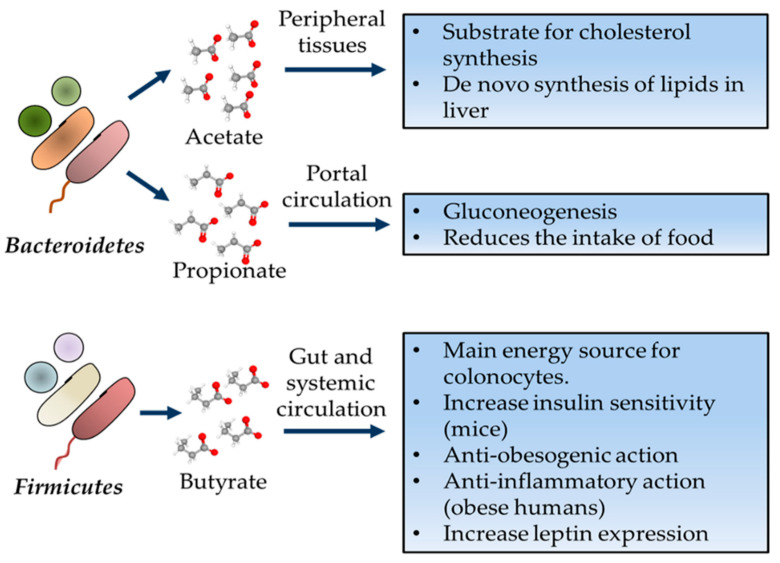
Functions of short-chain fatty acids (SCFAs) in the human body. Acetate, propionate, and butyrate are produced by colon microbiota and have an important paper in human health.

The interplay between diet, gut microbiota, and host energy metabolism are linked to short-chain fatty acids (SCFAs), the end products of bacterial metabolism of dietary undigested polysaccharides, which are acetate and propionate produce by Bacteroidetes and butyrate produced by Firmicutes [23,98]. Metabolic functions of main SCFAs are displayed in Figure 4.

**Figure 5:**
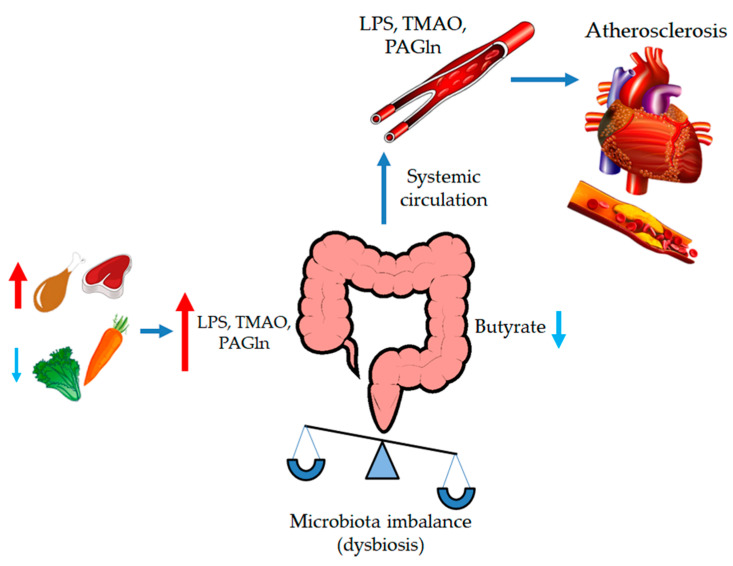
Microbiota dysbiosis promotes atherosclerosis formation. An obesogenic diet is rich in meat-derived choline and poor in fiber and complex carbohydrates. This type of diet promotes alteration in gut microbiota, and in consequence, affects intestinal permeability promoting bacterial translocation and increases in lipopolysaccharide (LPS), including trimethyl-amine (TMA) production, which is a precursor of TMAO. The presence of these molecules in peripheral tissues promotes inflammation and cardiac tissue development of atherosclerosis. Dysbiosis also decreases the number of butyrate-producer bacteria, reducing its protective and beneficial effects.

Patients with obesity present an overgrowth of *Lactobacillus*, *Escherichia coli*, and *Faecalibacterium*, among other bacteria. This “obese microbiota” showed an increased ability to extract calories from the diet [99]. Differences between the microbiota of obese and healthy individuals have been described, linking lower numbers of Bacteroidetes and the abundance of Firmicutes with obesity [100]. However, this observation remains controversial [99]. Moreover, obese patients present with less microbial diversity compared to lean subjects [96,101]. Additionally, obesogenic diets are poor in complex carbohydrates, mainly found in vegetables and fruits. This lack of dietary fiber leaves gut bacteria without a substrate to produce the end products of fermentation (SCFAs). Growing evidence has shown that a reduction in the levels of gut butyrate generates local inflammation and foam cell formation, contributing to gut barrier disruption and favoring bacterial translocation including mobilization of lipopolysaccharides (LPS), trimethylamine N-oxide (TMAO) and phenylacetyl glutamine (PAGIn) [102]. As described in Figure 5, LPS and TMAO in general circulation induce systemic inflammation; leading to macrophage activation and favoring formation of atherosclerosis plaques [103,104].

Along with SCFAs, TMAO is a key molecule derived from the bacterial metabolism that plays an important role in the development of CVD. TMAO is generated from dietary choline, L-carnitine, and betaine, which are metabolized by bacteria to produce trimethyl-amine (TMA), and further converted to TMAO in the liver. High levels of circulating TMAO induced the activation of NF-κB, increasing the expression of genes with pro-inflammatory effects, which increased oxidative stress. TMAO contributes to platelet hyperreactivity and thrombosis [105]. In addition, TMAO has been associated with coronary artery disease, prolongation of the hypertensive effect of Ang II, and poor prognosis in chronic and acute HF [106]. The extensive role of TMAO in CVD is reviewed in the article by Yang et al. [105].

Additionally, butyrate can impact the prevention of CVD by increasing the expression of ABCA1 in macrophages and, in consequence, promote apoA-I-mediated cholesterol efflux and increasing PPARγ levels [107]. In addition, butyrate has been shown to regulate reverse cholesterol transport via stimulating apoA-IV-containing lipoprotein secretion. Some butyrate-producing bacteria are *Roseburia intestinalis, Butyrivibriocrossotus*, and *Faecalibacterium prausnitzii,* which are almost depleted in atherosclerotic CVD patients [102,108]. In healthy individuals, these types of bacteria are present in high amounts [109,110].

Another microbiota mechanism involved in CVD development is their effect on arterial hypertension. Evidence showed that gut dysbiosis induces and maintains high levels of blood pressure through the SCFAs effects, systemic inflammation, and vasoactive metabolites, including serotonin, dopamine, norepinephrine, p-cresol sulfate, indoxyl sulfate, and TMAO [96,111]. Yang et al. [24] evaluated the dysbiosis effect in a rat model of hypertension and in a small cohort of hypertensive patients. They observed a significant decrease in microbiota diversity and abundance in rats with hypertension, accompanied by an increase in Firmicutes/Bacteroidetes ratio and low levels of acetate and butyrate-producing bacteria. In addition, in the small cohort of patients with hypertension, they described a similar dysbiosis. After patients received oral minocycline, the microbiota balance was reestablished, and the ratio Firmicutes/Bacteroidetes was reduced [112].

Some studies support the causality role of microbiota imbalance in hypertension; dysbiotic fecal samples were transferred from hypertensive patients into normotensive mice, resulting in elevated blood pressure in rodent recipients [112]. Also, SCFAs interact with host cells via G protein-coupled receptors (GPCRs), including Gpr41 and Olfr78; where activation of Gpr41 leads to hypotension, and Olfr78 increases blood pressure [112]. These results demonstrate that high blood pressure is associated with gut microbiota changes.

Additionally, the interaction between host and microbiota impacts proteins related to epithelial, lipid metabolism, and central nervous system functions. Zhernakova et al. [113] evaluated the association between plasma concentrations of 92 CVD-related proteins in patients (*n* = 1500); the results showed a microbial association in 41 proteins. Genetic and microbial factors collectively explain approximately 76% of the inter-individual variation, revealing succinct evidence for the microbial role in CVD.

Other bacterial metabolites like succinate can cause cardiac hypertrophy in murine models, whereas its levels are increased in patients with hypertrophic cardiomyopathy [96]. Troseid et al. [114] described the association of TMAO with disease severity and survival of patients with chronic HF; the results showed an elevated plasma level of TMAO in patients (*n* = 155) with chronic HF compared with the control group. Approximately 50% of patients with the highest levels of TMAO died or received a heart transplant during 5.2 years of follow-up [115].

The field of microbiota and their relation with diseases is rapidly growing. The richness of the microbiome and their metabolites will raise more scientific evidence of their role in the development of obesity-related CVD and their possible manipulation to treat it. Table 1 summarizes the bacteria associated with CVD pathophysiology.

## 6. Biomarkers Proposed for Cardiovascular Diseases

Biomarkers are traditionally classified according to their intended use as screening, diagnostic or prognostic biomarkers. Precision, high sensitivity, and specificity are fundamental characteristics of an ideal biomarker. In 2009, the American Heart Association defined the criteria for the evaluation of new biomarkers for clinical use [21]. In Table 2, currently used and proposed biomarkers for the diagnosis of CVD are enlisted.

## 7. Cell Lines and Animal Models Used to Study Cardiac Alterations Associated with Obesity

Several cell lines and animal models have been used to study obesity. Similar to humans, obesity in certain animal models is associated with co-morbidities such as systemic insulin resistance, diabetes, and hypertension [20]. Table 3 summarizes the most common cell lines and animal models used in the development of lipotoxic cardiomyopathy.

## 8. Therapeutic Approaches for Obesity-Linked CVD

### 8.1. Renin-Angiotensin-Aldosterone Inhibitors

Direct renin inhibition may be a promising antifibrotic therapy. It was reported that the oral renin inhibitor aliskiren has effects on collagen metabolism in cardiac fibroblasts and avoided myocardial collagen deposition in a non-hypertrophic mouse model of myocardial fibrosis, suggesting that aliskiren might be an effective therapy in HFpEF [145]. Aliskiren was approved by the Food and Drug Administration (FDA) in 2007 as antihypertensive. In addition, aliskiren might have renoprotective effects, which are independent of its blood pressure lowering effects in individuals with hypertension, T2D, and nephropathy [146].

Angiotensin-converting enzyme inhibitors (ACEIs) such as lisinopril, enalapril, and captopril, prevent the conversion of inactive AngI into active AngII, and have been used effectively in the treatment of several human diseases, including hypertension, congestive HF, coronary artery diseases, and diabetic nephropathy [147,148,149,150]. For example, lisinopril can regress myocardial fibrosis and improve LV diastolic function, while enalapril antagonizes the activation of the TGF-β signaling pathway [147,149].

Aldosterone plays a key role in the regulation of blood pressure and plasma sodium levels, promoting sodium retention in the renal tubules. In animal models, heart interstitial and perivascular fibrosis is caused by chronic administration of aldosterone and high salt intake. Treatment with spironolactone, an aldosterone antagonist, has been shown to prevent the increase in total and interstitial collagen in the heart [151,152].

### 8.2. Nutraceutics and Supplements

Recent data have shown the usefulness of a nutraceutical supplementation approach for the prophylaxis and treatment of heart disease. In particular, a large amount of clinical data showed the protective effect of polyunsaturated fatty acids in CVD. Therefore, linoleic acid (LA, 18: 2, omega-6) and linolenic acid (ALA,18:3, omega-3), called essential fatty acids, must be included in the diet [153]. ALA in combination with pirfenidone has shown an enhanced antioxidant effect [154].

Epigallocatechin-3-gallate (EGCG) is the most abundant and powerful catechin in green tea. In rats, EGCG inhibits cardiac fibroblasts proliferation and improves cardiac hypertrophy via inhibition of oxidative stress. EGCG decreased collagen synthesis and fibronectin expression in rat cardiac fibroblasts induced by Ang II. Moreover, it markedly ameliorated the excessive expression of CTGF and cardiac fibrosis via the blockage of NF-κB signaling pathway in hypertrophic stimulation. However, a high dose of EGCG results in cardiac collagen synthesis and aggravates cardiac fibrosis in mice [155].

Quercetin is the most widely distributed flavonoid, is abundant in red onions, citrus fruits, grains, among others. In rats being fed a Western diet supplemented with quercetin, cardiac remodeling was prevented by inhibition of the NF-κB signaling pathway and by the promotion of Nrf-2 and its downstream molecules. Luteolin is a flavone abundant in thyme, onion, broccoli, and cauliflower. Inhibits cardiac fibroblasts proliferation through reduction in oxidative stress in vitro. Luteolin blocks NOX2 and NOX4 in cardiac hypertrophy, thereby decreases the phosphorylation of JNK and TGF-β1 expression and reduces cardiac fibrosis [155].

Apigenin is another flavonoid; it modulates the activity of PPARγ and glucose/lipid metabolism. Also, apigenin attenuates myocardial injury induced by isoproterenol through the regulation of Pparγ in diabetic rats. Apigenin also mitigates cardiac remodeling by inhibition of oxidative stress, NF-κB pathway, and apoptosis, and through the reduction in cardiac fibrosis in streptozotocin-induced diabetic cardiomyopathy [155].

Isoflavones, such as genistein and daidzein, are found in soybeans and have beneficial antifibrotic effects on cardiac remodeling. Genistein inhibits TGF-β1-induced proliferation, as well as collagen production and myofibroblast transformation. Anthocyanins such as malvidin-3-glucoside, delphinidin-3-glucoside, cyanidin-3-glucoside, petunidin-3-glucoside, and peonidin-3-glucoside extracted from grape skins have protective effects on the ischemia/reperfusion. The flavanone hesperidin, presented in citrus peels, showed beneficial cardiovascular effects in animal models due to its antioxidative and antiapoptotic properties and increased Nrf-2 mRNA expression protecting the heart of aged rats. Naringenin has been shown to possess protective effects on lipid metabolism. In H_2_O_2_-treated cardiomyoblasts, naringenin treatment decreased stress-induced apoptotic cell death and lipid peroxidation and increased the reduced glutathione [155].

### 8.3. Histone Deacetylases (HDACs) Inhibitors

Several studies have demonstrated that HDACs are dysregulated in cardiac fibrosis, and many reports of preclinical studies showed the important role of HDAC inhibitors in the treatment of cardiac fibrosis. Valproic acid (VPA) attenuated cardiac hypertrophy and fibrosis through acetylation of the mineralocorticoid receptor in spontaneously hypertensive rats [156] and avoided right ventricular hypertrophy. Also, it was reported to reduce Ang II-induced pericyte-myofibroblast transdifferentiation and cardiac fibrosis by HDAC4-dependent phosphorylation of ERK [156].

MPT0E014, a pan-HDAC inhibitor (HDACI), downregulated TGF-β and Ang II type I receptor (At1r) in isoproterenol-induced dilated cardiomyopathy, whereas Trichostatin A (TSA) induced myocardial repair and prevented cardiac remodeling through c-kit signaling. TSA was also founded to reverse atrial fibrosis and reduced the incidence of arrhythmia without affecting the level of Ang II. Lyu et al. demonstrated that Class I HDAC inhibitor CI-994 reduced atrial fibrillation and fibrosis [156]. Mocetinostat, a selective Class I HDACI, inhibits the up-regulation of HDAC1 and HDAC2 in an animal model of congestive HF by reversing myofibroblast phenotype and increasing apoptosis, reducing fibrosis, and improving cardiac function by potentially blocking Akt signaling and inducing cell cycle arrest via p21/p53 [157]. In addition, the treatment with mocetinostat in cardiac fibroblasts from failing hearts showed decreased Col III, fibronectin, TIMP1, and IL-6-mediated STAT3 signaling [158]. Mocetinostat was also able to decrease Ang II-induced fibrosis [158], and attenuate IL-6/STAT3 signaling and decrease interstitial fibrosis and scar size in ventricular tissue in an animal model of HF [156]. MGCD0103 is another selective class I HDACI, which causes inhibition of Ang II-induced cardiac fibrosis by controlling the differentiation of bone marrow-derived fibrocytes. Inhibition of class I HDACs with an apicidin derivative was found to prevent cardiac hypertrophy and failure in preclinical studies. Inhibition of HDAC6 by tubacin, decreased TGF-β1-induced myofibroblast markers and reduced cardiac fibrosis [156]. The specific HDAC3 inhibitor, RGFP966, prevented diabetic cardiomyopathy reducing cardiac dysfunction, hypertrophy, and fibrosis; avoiding the elevation of phosphorylated ERK1/2 (an initiator of cardiac hypertrophy) in OVE26 mice diabetic hearts [159].

### 8.4. Antioxidative Stress Therapies

Oxidative stress and fibrosis are involved in cardiac remodeling and failure. Allopurinol was shown to decrease myocardial oxidative stress and improve diastolic dysfunction in Ang II-induced hypertensive mice. Furthermore, allopurinol prevented cardiac fibrosis through modulation of the TGF-β1/SMAD signaling pathway [160].

In streptozocin-induced diabetic mice, resveratrol mitigated oxidative levels, and interstitial fibrosis. Meanwhile, in primary cultured mouse cardiac fibroblasts, it prevented myofibroblast differentiation through the suppression of ROS/ERK/TGF-β/periostin pathway [161].

Curcumin administration was able to suppress the deposition of Col I and Col III in the heart tissue of diabetic rats, accompanied by a reduction in TGF-β1 production, suppression of TβR II levels, and SMAD2/3 phosphorylation, and increased SMAD7 expression. Similar effects were found in human cardiac fibroblasts exposed to high glucose [162].

Dysfunction of cardiac mitochondria is a hallmark of HF and causes oxidative stress. Therefore, special emphasis has been placed on vitamin E (α-tocopherol), vitamin C, and Co-enzyme Q10, which were found to have antioxidant activity in experimental models and patients with HF. However, in some clinical trials, antioxidants have shown disappointing results, except for vitamin c, and coenzyme Q10 [163].

### 8.5. Transforming Growth Factor-β Inhibitors

Pirfenidone and tranilast are two clinically-approved drugs, which have effects on inflammation and other fibrotic pathways. Furthermore, both drugs inhibit TGF-β signaling and have recently garnered interest as a potential treatment for cardiac fibrosis [164]. Pirfenidone inhibited TGF-β1 expression and the pro-fibrotic effects of TGF-β signaling, decreasing expression of Smad-7, TIMP-1, PAI-1, Col I, Col III, and Col IV [165]. In the short-term, pirfenidone and spironolactone treatment reversed cardiac as well as renal fibrosis and reduced the increased diastolic stiffness without normalizing cardiac contractility or renal function in streptozotocin-diabetic rats [166]. In addition, pirfenidone prevented myocardial steatosis and fibrosis in a mouse model of nonalcoholic steatohepatitis (NASH) by overexpressing PPARα, PPARγ, ACOX1, and CPT1A protein levels and decreasing Timp1, Col I, Col III mRNA levels [59]. Based on the fact that pirfenidone might be a promising agent for the treatment of CVD, the PIROUETTE (Pirfenidone in patients with HF and preserved left Ventricular Ejection fraction) trial was designed as a randomized, double-blind, placebo-controlled phase II trial to evaluate the efficacy and safety of 52 weeks of treatment with pirfenidone in patients with chronic HFpEF [167].

Regarding tranilast, it suppresses TGF-β expression and activity, inducing downregulation of collagen production in fibroblasts. In multiple animal models of cardiomyopathies, including experimental diabetes in rats, tranilast was reported to reduce myocardial fibrosis [164]. For instance, in streptozotocin-induced diabetic (mRen-2) 27 rats, tranilast attenuated cardiac matrix deposition by reducing phospho-Smad2 levels [168]. In a similar model, tranilast improved LV systolic and diastolic function without affecting SMAD phosphorylation but attenuated TGF-β-induced p44/42 MAPK phosphorylation [168]. The antifibrotic effects of tranilast were associated with an inhibition of TGF-β signaling and suppression of the infiltration of inflammatory cells, including monocytes and macrophages. Furthermore, mRNA levels of TGF-β1, plasminogen activator inhibitor 1 (PAI-1), MCP-1, IL-6, pro-collagens were decreased, as well as myocardial fibrosis and collagen accumulation in deoxycorticosterone acetate/salt hypertensive rats receiving tranilast. Similar results were found in renovascular hypertensive rats and hypertensive (mRen-2) 27 rats. Interestingly, in these studies, the inhibition of cardiac fibrosis by tranilast is independent of changes in blood pressure, suggesting a direct effect on cardiac fibrosis, with potential for HF treatment [168].

## 9. Conclusions

In this review, we have described the pathophysiologic mechanisms involved in obesity-related CVD, as well as the role of PPARs, epigenetic modifications, and gut microbiota dysbiosis associated. It has become clear that hyperglycemia, as well as excess FFAs, and triglyceride levels promote WAT dysfunction, leading to an altered expression of pro-inflammatory cytokines, adipokines, and hormones, which activate pathological processes, such as oxidative stress and inflammation on WAT and cardiac tissue. In obesity, the renin-angiotensin-aldosterone system (RAAS) is activated, causing amplification of inflammation and structural remodeling, thus inducing cardiac and vascular damage, as well as other structural alterations leading to cardiac dysfunction. This review also provides information related to biomarkers currently in use and those proposed as diagnostic biomarkers of CVD, together with in vitro and animal models commonly used in CVD. In addition, therapeutic treatments for CVD were examined. Therefore, this review was conceived to provide an update of knowledge related to CVD associated with obesity, to improve understanding of the main pathological mechanisms involved, and to resume the potential therapeutic strategies available.

## Figures and Tables

**Table 1:** Bacteria involved in CVD development.

Type of Bacteria	Description
*Phascolarctobacterium, Proteus mirabilis* and Veillonellaceae	Propionate/acetate producers with a positive correlation between obesity and their augmented abundance in HFD-fed rats [116]
*Roseburia intestinalis* and *Faecalibacterium prausnitzii*	Reduced in diabetic patients [117]
Prevotellaceae and Archaea	H2-producing and utilizing bacteria accelerated fermentation and increased SCFAs production, and high-energy uptake [118]
*Escherichia coli, Klebsiella spp., Enterobacter aerogenes, Ruminococcus gnavus*, and *Eggerthella lenta*	Abundant in patients with atherosclerotic CVD compared with healthy subjects [119]
*Campylobacter, Shigella, Salmonella*, and *Yersinia enterocolitica*	Pathogenic bacteria colonizing the gut of patients with chronic HF [120]
*Escherichia/Shigella*	Abundancy in decompensated HF vs. compensated patients [121]
*Turicibacter, Roseburia, Lachnospira*, and *Romboutsia*	Positive relation with high levels of serum triglyceride, cholesterol, and low-density lipoprotein; and negative relation with the serum high-density lipoprotein in HFD-fed rats [121]
*Akkermansia muciniphila*	Decreased amounts in obesity and diabetic patients vs. healthy individuals; higher quantity is associated with improvement in cardiac metabolic parameters in obesity [122]
Lactobacillales, *Bacteroides* and *Prevotella*	Higher amounts of Lactobacillales and lower levels of Bacteroides and Prevotella has been observed in patients with coronary artery disease [123]
*Prevotella*	Abundant levels in patients with hypertension compared with healthy controls [124]
Lactobacillales, *Collinsella*, Enterobacteriaceae, and *Streptococcus* spp	Levels altered in patients with atherosclerotic CVD [125]
*Veillonella spp* and *Streptococcus* spp	Presented in atherosclerotic plaques in human patients. Correlated with their abundance in the oral cavity [126]

HFD: high-fat diet, SCFAs: short-chain fatty acids, CVD: cardiovascular disease, HF: heart failure.

**Table 2:** Biomarkers associated with CVD.

Biomarker	Functions	Disease Associated
ANP and BNP	ANP and BNP are secreted by cardiac cells during HF to counteract the onset of volume and pressure overload through their vasodilator and natriuretic effects	ANP and BNP are currently utilized as biomarkers for HF and myocardial infarction. Especially for two specific HF categories, HFmrEF and HF with HFpEF [21,127]
TnI and TnT	Troponins regulate calcium-mediated interaction between actin and myosin, thus are related to myocardial contractility	TnI and TnT are currently used as necrosis markers because their serum levels may be predictive for cardiovascular death in subjects with myocardial infarction and HF. They are proposed as biomarkers for diabetic cardiomyopathy [21,127]
sST2	sST2 is related to inflammatory and immune processes	sST2 is a cardiac biomarker cleared by the FDA for prognosis and diagnosis of chronic HF [21,127]
FABP4	FABP4 plays an essential role in the development of insulin resistance and atherosclerosis	FABP4 is a potent biomarker of FAs metabolic alterations and CVD [128]
FABP3	FABP3 transports FAs from the plasma membrane to mitochondria for β-oxidation	FABP3 might be a suitable diagnostic tool in systolic dysfunction, hypertrophic and dilated cardiomyopathy, including HF [127]
PIIINP	PIIINP is an indicator of extracellular matrix turnover	PIIINP is proposed as a marker of early LV dysfunction in patients with insulin resistance, as well as patients with HF with HFpEF [21,127]
Galectin-3	Galectin-3 is locally secreted by activated macrophages and fibroblasts, and it has a pro-fibrotic action	Galectin-3 has been proposed as a good prognostic biomarker of LV systolic dysfunction and HF in diabetic patients, as well as an indicator of cardiac tissue remodeling and fibrosis [21,127]
Adiponectin	Adiponectin is a cardioprotective agent	Adiponectin has been proposed as a biomarker of HF [20]
NGAL	NGAL functions as an inflammatory regulator of the innate immune system	NGAL is a promising diagnostic biomarker of CVD (atherosclerosis, acute coronary syndrome, stable coronary artery disease, and HF) [129]
IGFBP-7	IGFBP-7 is a modulator of insulin receptor activity and signaling	IGFBP-7 is a promising biomarker for collagen deposition, fibrosis, and cardiac hypertrophy in diabetes, as well as for diastolic dysfunction and HF [127]

ANP and BNP: natriuretic peptides A and B, HF: heart failure, HFmrEF: mid-range ejection fraction, HFpEF: preserved ejection fraction, TnI: troponin I, TnT: troponin T, sST2: soluble suppression of tumorigenesis 2, FDA: food and drug administration, FABP4: fatty acid-binding protein 4, FABP3: Fatty acids binding protein 3, FAs: fatty acids, PIIINP: pro-collagen type III aminopeptide, LV: left ventricle, NGAL: neutrophil gelatinase-associated lipocalin, CVD: cardiovascular disease, IGFBP-7: insulin-like growth factor binding protein-7.

**Table 3:** Cell lines and animal models of lipotoxicity.

Representative Model	Characteristics	Representative Treatments	Cardiac Phenotype Reported
Primary cardiomyocytes	Neonatal primary cardiomyocytes from one- to three-day-old wild type mice	Treated with palmitate for 24 h	Developed hypertrophy, inflammatory cytokines up-regulation, and oxidative stress [130]
AC16 cells	Human adult ventricular cardiomyocytes	Treated with palmitate for 16 h	Apoptosis [131]
HL-1 cells	Murine HL-1 cardiomyocytes	Treated with fatty acids for various time periods	Developed apoptosis and necrosis [132]
H9c2 cells	Cell line derived from an embryonic rat heart ventricle	Treated with palmitate for 24 h	Exhibited hypertrophy, up-regulation of inflammatory cytokines, and increased oxidative stress [130]
C57BL/6J mice	Susceptible to diet-induced obesity, T2D, and atherosclerosis. Deletion in nicotinamide nucleotide transhydrogenase (Nnt) exons 7–11	Sixteen weeks with a high-fat/high-carbohydrate diet consisted of 60% kcal from fat food and drinking water with 42 g/L of carbohydrates (55% fructose and 45% sucrose)	Male mice exhibited systemic insulin resistance, myocardial steatosis with inflammatory foci, hypertrophy, and fibrosis [59]
CD1 mice	Albino mice	Eight weeks with a Western diet with 42% total fat, 12.8% saturated fat, and 30% sucrose	Male mice showed impaired cardiac systolic and diastolic function, myocardial inflammation, and fibrosis [133]
C57BL6J db/db mice	The db/db mouse has a point mutation in the leptin receptor gene.	Exhibited rapid weight gain when fed a regular chow diet, analyzed at 6 and 12 months of age	Female mice exhibited an increase in blood pressure, both male and female developed hyperglycemia, hypertrophic ventricular remodeling, and diastolic dysfunction with HFpEF, cardiomyocyte hypertrophy, and interstitial fibrosis [134]
Wistar rats	Its longevity and high rate of spontaneous tumors make it an ideal choice for aging studies.	Six weeks with HFD: 33.5% fat	Male rats showed cardiac hypertrophy; cardiac weight, cardiac fibrosis, and inflammatory markers were increased [135]
Sprague-Dawley rats	Albino rats	Forty-eight weeks with fructose and fat: 60 kcal/100 kcal saturated fat with 10% fructose	Male rats developed severe obesity, symptoms of metabolic syndrome, systemic insulin resistance, intramyocardial lipid accumulation, and cardiac hypertrophy [136]
ZDF rats	Rats develop obesity and insulin resistance at a young age	Maintained on RMH-B rat chow	Male rats showed an increase in cardiomyocyte size, LV performance, also developed perivascular fibrosis and cardiac hypertrophy [137]
ZSF1 rats	Two different leptin mutations (fa and facp)	They become hyperphagic and develop obesity. Analyzed at 26 weeks of age.	Female and male rats showed severe dyslipidemia without hyperglycemia, also displayed diastolic dysfunction, cardiac hypertrophy, and fibrosis [138]
Spontaneously hypertensive rats (SHRs)	Hypertension starts to develop at five to six weeks of age	Twelve weeks with HFD: 60% fat, 20% carbohydrate, and 20% protein	Male rats showed hyperglycemia, dyslipidemia, showed a constellation of LV diastolic dysfunction, and myocardial fibrosis [139]
Hamsters	Normal Hamsters	Six weeks with HFD: Sucrose (162.58 g/kg), Soybean oil(162.58 g/kg), among others	Male hamsters developed cardiac fibrosis [140]
New Zealand White rabbits	Rabbits have a genetic deviation called albinism	Twelve weeks with standard rabbit chow with 10% added fat (6.7% corn oil and 3.3% lard)	Female showed elevated LV weight, interstitial and perivascular collagen, fibrosis in coronary vessels, as well as accumulation of collagen in the cardiac interstitium [141]
Sheeps	Healthy obese Sheeps	Four months with high-energy soybean oil (2.2%), molasses, fortified grain, and maintenance hay	Sheeps exhibited increased LA volume, inflammatory infiltrates, and fibrosis [142]
Lee-Sung minipigs	Healthy obese minipigs	Six months with HFD (3786 Kcal/ kg, metabolic energy)	Males and females showed augmented heart weight, interstitial and perivascular fibrosis, cardiac lipid accumulation and increased oxidative stress [143]
Bama miniature pigs	They have metabolic similarities to humans: lack of brown fat, and proportional organ sizes and cardiovascular systems	23 months fed with a high-fat, high-sucrose diet (37% sucrose, 53% control diet, and 10% pork lard)	Pigs developed symptoms of metabolic syndrome and showed cardiac steatosis and hypertrophy. Insulin levels and heart weight were increased [127]
Mongrel dogs	Healthy dogs	Six weeks with a standard diet supplemented with 6 g/kg of rendered pork fat; 21,025 kJ/day (27% carbohydrate, 19% protein, and 53% fat)	Male dogs showed increased fasting insulin and markedly reduced insulin sensitivity, including a reduction in left ventricular function [144]

AC16: cardiomyocyte cell line, HL-1: cardiac muscle cell line, H9C2: rat cardiomyoblast cell line, C57BL/6J: commonly called Black 6 mouse, T2D: type 2 diabetes, HFpEF: preserved ejection fraction, HFD: high-fat diet, ZDF: Zucker diabetic fatty, RMH-B: standard rat diet chow, LV: left ventricle.
